# Bronze Age meat industry: ancient mitochondrial DNA analyses of pig bones from the prehistoric salt mines of Hallstatt (Austria)

**DOI:** 10.1186/s13104-018-3340-7

**Published:** 2018-04-13

**Authors:** Sabine E. Hammer, Barbara Tautscher, Erich Pucher, Kerstin Kowarik, Hans Reschreiter, Anton Kern, Elisabeth Haring

**Affiliations:** 1 Institute of Immunology, Department of Pathobiology, University of Veterinary Medicine Vienna, Veterinaerplatz 1, 1210 Vienna, Austria; 2 Central Research Laboratories, Museum of Natural History Vienna, Burgring 7, 1010 Vienna, Austria; 3 1st Zoological Department, Archaeozoological Collection, Museum of Natural History Vienna, Burgring 7, 1010 Vienna, Austria; 4 Prehistoric Department, Museum of Natural History Vienna, Burgring 7, 1010 Vienna, Austria; 5 Department of Integrative Zoology, University of Vienna, Althanstrasse 14, 1090 Vienna, Austria

**Keywords:** Hallstatt, Bronze Age, *Sus scrofa*, Ancient DNA, Mitochondrial DNA, Phylogeographic network

## Abstract

**Objective:**

In the Bronze Age Hallstatt metropolis (‘Salzkammergut’ region, Upper Austria), salt richness enabled the preservation of pork meat to sustain people’s livelihood suggesting an organized meat production industry on a yearly basis of hundreds of pigs. To pattern the geographic and temporal framework of the early management of pig populations in the surrounding areas of Hallstatt, we want to gain insights into the phylogeographic network based on DNA sequence variation among modern pigs, wild boars and prehistoric (likely) domestic pigs.

**Results:**

In this pilot study, we successfully adapted ancient DNA extraction and sequencing approaches for the analysis of mitochondrial DNA sequence variation in ten prehistoric porcine teeth specimens. Minimum-spanning network analyses revealed unique mitochondrial control region DNA haplotypes ranging within the variation of modern domestic pig and wild boar lineages and even shared haplotypes between prehistoric and modern domestic pigs and wild boars were observed.

**Electronic supplementary material:**

The online version of this article (10.1186/s13104-018-3340-7) contains supplementary material, which is available to authorized users.

## Introduction

The UNESCO World Heritage Hallstatt-Dachstein/Salzkammergut, located in the eastern Austrian alps, represents one of the most important prehistoric production centres in Europe. Underground salt mining at depths of up to 170 m is attested as far back as 1500 years BC [1, 2]. Due to the high salt concentrations inside the prehistoric mining galleries, the remains of the extraction activity have been perfectly preserved [3, 4]. The size of the mining areas and the amount of mining waste evidence production on a large scale as well as a highly structured and technologically well-developed organization [1, 2]. Set in an alpine environment, with the production and settlement areas located in a narrow valley at an elevation of 1000 m above sea level (MASL), the prehistoric mining community was nonetheless able to meet the exacting demands of the large scale production activity. In Bronze Age, economic activity encompassed not only salt extraction, but also the production of cured meat, mainly from pig, on a very large scale. A special butchering technique, documented through thousands of pig bones, as well as facilities for curing the meat, 8 log basins with the capacity to hold up to 200 butchered pigs, attest to the developed organization and scale of this meat production industry. Currently archaeological data indicates that the meat of several hundred pigs was annually processed and cured in the *Hallstatt High Valley* [5–7]. The archaeozoological analysis of the bone inventory evidences a well organised system based on the breeding of animals for the “meat industry” and the transport of meat from the animal breeders to the *Hallstatt High Valley*. Morphological differences point to different areas of the pigs’ origin, to the North along the Traun river and to the Southeast towards the Styrian Salzkammergut [8].

The large scope of the project should delineate the geographic and temporal framework of the early management of pig populations in the surrounding areas of Hallstatt and allow to estimate the spatial extent of the catchment areas. These attempts should help to answer the questions which husbandries delivered pigs to Hallstatt and whether or not a natural pig breeding monopoly existed during the Hallstatt period. Ancient DNA elucidates phylogenetic relationships of pigs allowing insights into prehistoric farming practices as well as adaptation and domestication of pigs [9–13]. The high abundance of porcine teeth remnants at the *Hallstatt High Valley* initiated this pilot study to assess the suitability of this material for molecular genetic analyses. The study gained first insights into genetic variation in a neutral DNA marker sequence, the mitochondrial (mt) control region (CR) and thus will pave the way for deeper molecular analyses by nuclear marker assessment.

## Main text

### Methods

#### Animals and sample collection

The studied material comprises 10 mandibular cuspids (fangs), mainly from castrated males, excavated in 1939 and 1993/94 in the *Hallstatt High Valley* (‘Salzkammergut’ region, Upper Austria) (Table 1). The town of Hallstatt is situated at 47.56° North latitude, 13.65° East longitude and 514 MASL. The analysed specimens derived from an thick layer of animal bone assemblage being radiocarbon dated to the 13th/12th century BC, the Late Bronze Age [1, 2]. These animal bones are part of the Archaeozoological Collection at the Natural History Museum Vienna (NHMW), Austria. Detailed information on prehistoric pig specimens as well as modern *Sus scrofa* and Suinae taxa of the present study is given in Table 1.

**Table 1:** List of mitochondrial DNA sequences of prehistoric and modern domestic pigs, wild boars and Suinae obtained in the present study or downloaded from GenBank

Accession no	Tree/network label	Location	Species	Status	Breed	Source
*MG926393*	*H45-1 (AT)*	*Austria*	*Sus scrofa f. domestica*	*Prehistoric domestic*	–	*This study*
*MG926394*	*HoN-4 (AT)*	*Austria*	*Sus scrofa f. domestica*	*Prehistoric domestic*	–	*This study*
*MG926395*	*H405-5 (AT)*	*Austria*	*Sus scrofa f. domestica*	*Prehistoric domestic*	–	*This study*
*MG926396*	*H124-6 (AT)*	*Austria*	*Sus scrofa f. domestica*	*Prehistoric domestic*	–	*This study*
*MG926397*	*H288-7 (AT)*	*Austria*	*Sus scrofa f. domestica*	*Prehistoric domestic*	–	*This study*
*MG926398*	*H51-1 (AT)*	*Austria*	*Sus scrofa f. domestica*	*Prehistoric domestic*	–	*This study*
*MG926393*	*H117-21 (AT)*	*Austria*	*Sus scrofa f. domestica*	*Prehistoric domestic*	–	*This study*
*MG926393*	*H136-3 (AT)*	*Austria*	*Sus scrofa f. domestica*	*Prehistoric domestic*	–	*This study*
DQ379225	Saddleback (DE)	Germany	*Sus scrofa f. domestica*	Domestic	Angeln Saddleback	[29]
AY884775	Landrace-01 (FI)	Finland	*Sus scrofa f. domestica*	Domestic	Landrace	[9]
AY884748	Landrace-02 (NO)	Norway	*Sus scrofa f. domestica*	Domestic	Landrace	[9]
AY884746	Duroc (GB)	United Kingdom	*Sus scrofa f. domestica*	Domestic	Duroc	[9]
AY884779	Creole (FR)	France	*Sus scrofa f. domestica*	Domestic	Creole	[9]
DQ152846	Large White (EU)	Europe	*Sus scrofa f. domestica*	Domestic	Large White	[29]
AY884763	Large White (FR)	France	*Sus scrofa f. domestica*	Domestic	Large White	[9]
AY884785	Large White (DE)	Germany	*Sus scrofa f. domestica*	Domestic	Large White	[9]
AY884751	Linderodssvin (SE)	Sweden	*Sus scrofa f. domestica*	Domestic	Linderodssvin	[9]
AY884769	Piétrain (DE)	Germany	*Sus scrofa f. domestica*	Domestic	Piétrain	[9]
AY884764	Mangalica (HU)	Hungaria	*Sus scrofa f. domestica*	Domestic	Mangalica	[9]
DQ152879	Bamei (CN)	China	*Sus scrofa f. domestica*	Domestic	Bamei	[29]
DQ152886	Huzhu (CN)	China	*Sus scrofa f. domestica*	Domestic	Huzhu	[29]
DQ379162	Meishan (CN)	China	*Sus scrofa f. domestica*	Domestic	Meishan	[29]
DQ152868	Zang (CN)	China	*Sus scrofa f. domestica*	Domestic	Zang	[29]
HM747197	Wild boar (AT)	Austria	*Sus scrofa*	Wild	–	[29]
AY884664	Wild boar (DE)	Germany	*Sus scrofa*	Wild	–	[9]
DQ379236	Wild boar (BE)	Belgium	*Sus scrofa*	Wild	–	[29]
DQ379253	Wild boar-1 (FR)	France	*Sus scrofa*	Wild	–	[29]
DQ379244	Wild boar-2 (FR)	France	*Sus scrofa*	Wild	–	[29]
FJ236998	Wild boar (ES)	Spain	*Sus scrofa*	Wild	–	Fernandez AI (26-SEP-2008)
AY884672	Wild boar (NO)	Norway	*Sus scrofa*	Wild	–	[9]
AY884670	Wild boar (MK)	Mecedonia	*Sus scrofa*	Wild	–	[9]
AY884726	Wild boar (AM)	Armenia	*Sus scrofa*	Wild	–	[9]
DQ872938	Wild boar-1 (IR)	Iran	*Sus scrofa*	Wild	–	[9]
DQ872956	Wild boar-2 (IR)	Iran	*Sus scrofa*	Wild	–	[9]
AY884612	Wild boar (IN)	India	*Sus scrofa*	Wild	–	[9]
AY884661	Wild boar (ID)	Indonesia	*Sus scrofa*	Wild	–	[9]
DQ379262	Wild boar-1 (CN)	China	*Sus scrofa*	Wild	–	[29]
DQ379266	Wild boar-2 (CN)	China	*Sus scrofa*	Wild	–	[29]
AY884702	*S. scrofa papuensis*	Papua New Guinea	*Sus scrofa papuensis*	Wild	–	[9]
AY884708	*S. scrofa taiwanensis*	Taiwan	*Sus scrofa taiwanensis*	Wild	–	[9]
AY884705	*S. scrofa andamensis*	Andaman Islands (India)	*Sus scrofa andamensis*	Wild	–	[9]
KF952600	*Sus cebifrons*	Philippines	*Sus cebifrons*	Wild	–	Si T (10-DEC-2013)
KP789021	*Sus barbatus*	Southeast Asia	*Sus barbatus*	Wild	–	Zhang S (13-FEB-2015)
KF926379	*Sus verrucosus*	Indonesian	*Sus verrucosus*	Wild	–	Fan J (03-DEC-2013)

Specimens from Hallstatt are written in Italic underline. In column ‘Tree/Network label’, the ISO 3166 Countries Codes are given in brackets

#### DNA extraction

DNA extractions were performed in a clean room by obeying all standard routines for working with aDNA [14, 15] (For details see Additional file 1). Cleaning and decontamination of grinding bowls and balls was performed with *DNA*-*Away*^1^ after an ultrasonic bath followed by subsequent UV radiation. All post-PCR work was carried out in a separate laboratory. Extraction controls (buffers without sample) were performed to screen for contaminated extraction reagents. For each specimen, at least two independent DNA extractions were performed. The detailed protocol for DNA extraction is given in Additional file 1. Briefly, prior to the DNA extraction, the surface of each tooth was treated with 3% sodium hypochlorite and rinsed in nuclease free water^2^ for decontamination [16]. The dried teeth were crushed into small pieces and pulverized with a Retsch MM400 grinding mill.^3^ Next, 1 g tooth powder was decalcified three times by adding 4.5 ml *Decalcifier soft*^4^ (containing 25% EDTA) and rotating overnight incubation at 4 °C. Following centrifugation (Eppendorf Centrifuge 5430^5^), the supernatant was discarded and replaced by 4.5 ml fresh decalcifying solution. After decalcification, the powder was washed three times by adding 4.5 ml nuclease free water to remove all remains of EDTA. The rinsed powder was subjected to DNA extraction with the *Gen*-*ial All Tissue Kit*^6^ according to the manufacturer’s instructions for DNA extraction from bone to teeth. Finally, the DNA was dissolved in 30 µl nuclease free water (see footnote 2) and after concentration measurement (BioPhotometer D30, µCuvette G1.0) (see footnote 5), the DNA solutions were immediately aliquoted (5–10 µl) and stored at 4 °C (short term) or at − 20 °C for long term.

#### PCR amplification of the mitochondrial control region

A 721-basepair-long section of the mitochondrial control region was amplified using three PCR primer pairs that produce three overlapping amplicons, ranging from 343 to 401 base pairs (bp) in length (Additional files 2, 3 and 4). PCR was performed with Amplitaq Gold^®^ 360 DNA-Polymerase^7^ and PCR reactions were run on a Mastercycler Nexus (See footnote 5) by applying conventional thermal cycling conditions and touch-down protocols (Additional file 1). Failed PCR reactions were repeated with varying amounts of template DNA. Control PCR reactions were performed to screen for contaminated reagents: extraction control (buffers without sample) and non-template control with nuclease-free water instead of template. Finally, PCR products were purified with the QIAquick PCR Purification Kit (see footnote 2) and sequenced (both directions using the PCR primers) at Microsynth AG^8^ and LGC Genomics.^9^

#### DNA sequence analysis and phylogenetic reconstruction

BioEdit Sequence Alignment Editor (v7.2.5) was used for nucleotide sequence alignment and sequence editing [17]. The final dataset including previously published sequences (GenBank) had a length of 637 sites and comprised 42 sequences. Trees were calculated by the Neighbor-Joining (NJ) method [18], by Maximum-Likelihood (ML) as well as Bayesian inference (BI) using the software MEGA7 [19] for NJ and ML trees and MrBayes v3.2 [20] for BI trees. For the NJ analysis, the evolutionary distances were computed using the *p*-distance method [21] and are in the units of the number of base differences per site. The ML method was based on the Tamura 3-parameter model [22] with a discrete Gamma distribution to model evolutionary rate differences among sites (two categories: *G* = 0.58, *I* = 0.75). Parameters for BI analysis were as follows: lset nst = 2 rates = gamma [20]. FigTree v1.4.3 [23] was used to annotate the consensus tree produced by MrBayes. The unrooted phylogenetic network was constructed with PopART [24, 25] by applying the minimum spanning option.

### Results and discussion

Out of the ten samples analysed, seven allowed to determine the complete CR marker sequence (637 bp), whereas two were unsuccessful and one allowed to amplify fragment B (342 bp), only. Sequences obtained in the present study are deposited in GenBank (Accession no’s MG926393–MG926400, Table 1).

**Fig. 1:**
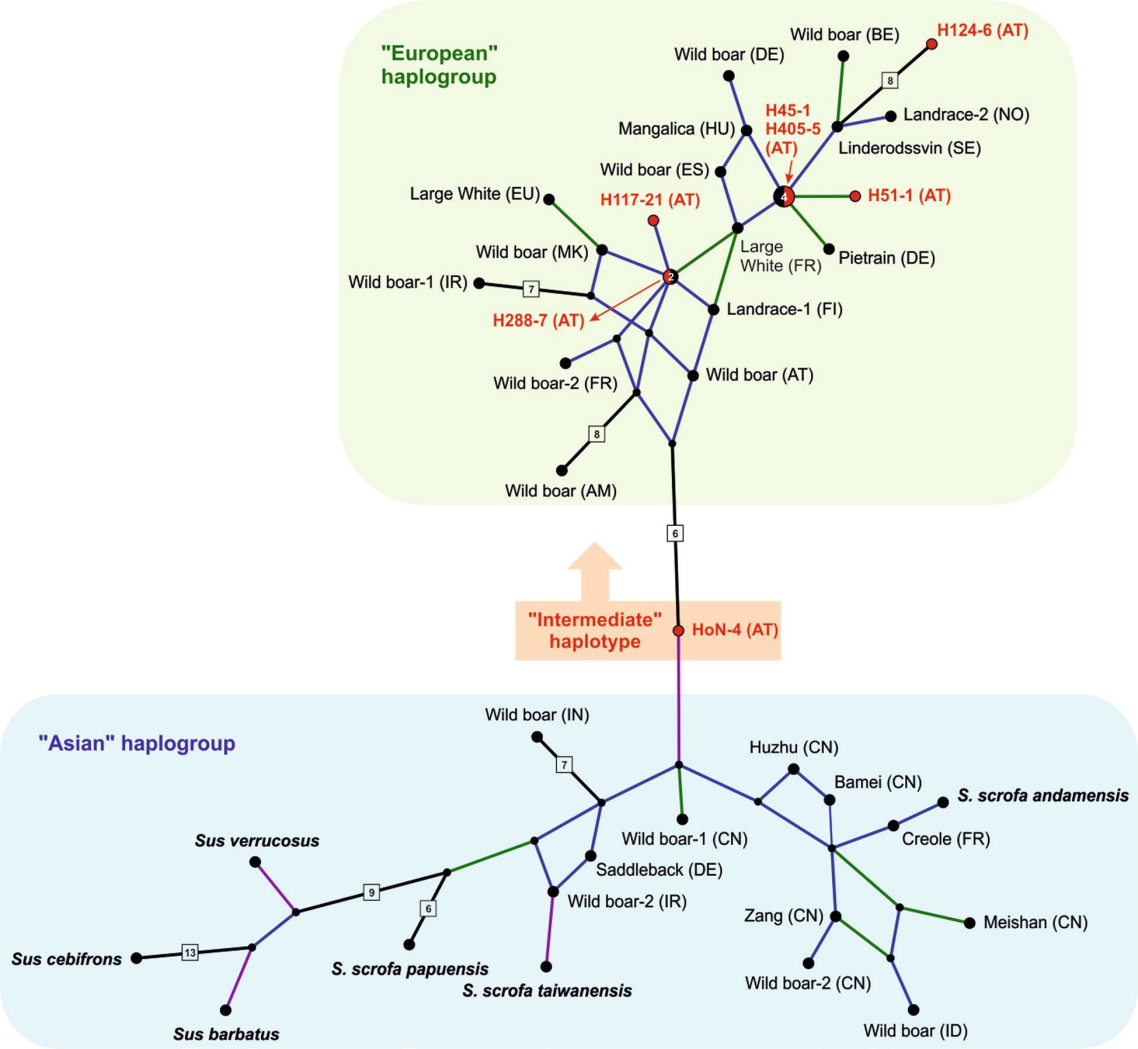
Minimum Spanning network illustrating the diversity of haplotypes of the mitochondrial control region of 35 modern wild and domestic Sus taxa and seven prehistoric domestic pigs (in red). The network is based on the 637 bp-alignment. The size of the circles is proportional to the number of individuals sharing the haplotype and the numbers are shown in the circles. Colour-coded connecting lines illustrate the number of nucleotide differences between haplotypes: 1 = blue, 2 = green and 3 = purple. Nucleotide differences between haplotypes greater than 3 are indicated by the boxed digits placed on the connecting lines. Detailed information on taxa and samples is given in Table 1 by defining the specimens’ status as domestic or wild and indicating the pig breed, if applicable. For information on shared haplotypes see main text

As can be seen in Additional file 5, PCR success is not strictly correlated with DNA concentrations e.g., one of the unsuccessful samples, H188-7 proved to have the highest DNA concentrations, while the sample with the lowest concentration H117-21 allowed to amplify all fragments. Unfortunately, the overall amount of DNA that could be extracted from each tooth was limited and thus only a few PCR trials were possible until the DNA was completely used up. Since DNA concentration seems to be not a reliable predictor of fragmentation it appears reasonable to further reduce the amplicon size. This will of course increase the effort necessary to obtain complete sequences, but, on the other hand, will allow to determine the sequence from a higher number of samples. Three samples (H45-1, H117-21, HoN-4) delivered sequences without any ambiguities, while in samples H405-5, H288-7, H51-1, and H124-6 several ambiguous sites were found, which could be interpreted as derived from post-mortem modifications. Almost all of them could be determined by repetition of PCR and subsequent sequencing as well as with the information from overlapping regions. Only in sample H124-6 two C/T ambiguities could not be resolved and are coded as Y in the alignment. The final alignment (637 bp) comprising 42 sequences of prehistoric and modern domestic pigs, wild boars and Suinae (Table 1) had 567 conserved and 35 parsimony informative sites. The *p*-distances among prehistoric domestic pigs ranged from 0.2 to 3.2% (average 1.1%), while distances within the in-group were up to 3.9% (average 1.6%) and between *S. scrofa* and the outgroup taxa distances ranged from 2.4 to 5.7% (average 3.7%) (Additional file 6). A minimum spanning network based on this alignment is roughly divided into two groups (Fig. 1). Interestingly, the “Asian” group includes sequences of Asian as well as European origin, as well as the outgroup taxa. In contrast, the “European” group exclusively consists of sequences derived from European breeds as well as prehistoric pigs from Hallstatt. One sequence from Hallstatt (HoN4) has an intermediate position between the two haplogroups. The inclusion of the outgroup sequences into the network illustrates that distances within *S. scrofa* are almost in the same range as between *S. scrofa* and the outgroup (see also Additional files 6, 7). A rooted NJ tree is given in Additional file 8 to alternatively illustrate the distances between sequences and shows the same overall topology as the ML and BI trees (support values of all analyses are included in the NJ tree in Additional files 8, 9). As the separation into two haplogroups is not well supported in this NJ tree, future analyses of additional markers should help to support this geographic pattern. Concerning the distribution of prehistoric samples in the network, there are two shared haplotypes harboured by prehistoric as well as modern pigs: H288-7 was identical with a Wild boar specimen from Norway to H45-1 and H405-5 shared the same haplotype with a British Duroc and the French Wild boar-1.

### Conclusions

There are three major outcomes: (1) The results indicate that the teeth are suitable material to obtain genetic information from prehistoric *Sus domestica* (*S. scrofa* f. domestica) from Hallstatt. Next steps are to test nuclear markers (e.g., nuclear DNA sequences of mitochondrial origin (numts) [26]; Y chromosome, *MCR1* [27]; SLA-DRB1 [28]) with the tooth material as well as bones to assess the potential success of genomic analyses. (2) The variety found in the mitochondrial marker sequence of prehistoric pigs is almost as high as found among present day *S. scrofa* (Wild boar and breeds). Although the data presented here can be considered only as first hints, they are in favour for the assumption that the Hallstatt pigs were derived from large herds and/or various husbandries. However, the placement of prehistoric pigs in the haplotype network and the phylogenetic tree do not allow to draw conclusions about their geographical origin and status (wild vs. domesticated). The results are in accordance with earlier findings implying repeated gene flow between wild boar and domestic breeds [9, 29, 30]. (3) With the exception of the intermediate haplotype, all prehistoric pigs from Hallstatt resemble haplotypes of the “European” group. Shared haplotypes between prehistoric and modern *S. scrofa* indicate that Hallstatt pigs did not represent an independent lineage, but seem to range within the variation of extant *S. scrofa*.

## Limitations

Limitations of this study were mainly due to the low number of prehistoric pigs that have been analysed and the fact that DNA sequence analyses in this pilot study are based on a single mitochondrial marker system. Moreover, the suitability of other bony material for obtaining genetic information from prehistoric pig specimens has to be proven in subsequent experiments. Future nuclear marker assessments will assist in drawing a clearer picture of prehistoric Hallstatt’s meat production and surrounding husbandries. However, any signal could be blurred by a substantial amount of gene flow between geographic regions as well as between wild boars and domesticated pigs.

## Additional files

**Additional file 1.** Ancient DNA extraction and Mitochondrial control region PCR. Detailed protocols for Ancient DNA extraction and PCR of mitochondrial control region.

**Additional file 2.** Map of the porcine mitochondrial DNA. Localisation of the 721 bp long control region (CR) fragment in the reference mitochondrial genome.

**Additional file 3.** PCR strategy for the 721-bp-long section of the mitochondrial Control Region (CR). The CR sequences were inferred using three PCR primer pairs that allow amplification of overlapping amplicons, ranging from 343 bp to 401 bp in length.

**Additional file 4.** PCR strategy for the 721-bp-long section of the mitochondrial Control Region (CR). Primers used to amplify three sections (A, B, C) of the mitochondrial control region.

**Additional file 5.** Detailed sequencing outcome for the 10 prehistoric porcine teeth specimens. For each specimen, DNA concentration and the number of obtained sequence reads for fragments A, B and C, are given.

**Additional file 6.** Phylogenetic Reconstruction I—estimates of evolutionary divergence between sequences. Distance matrix shows the number of base differences per site (*p* distances; Nei and Kumar 2000) between 42 DNA sequences (length of alignment 637 bp).

**Additional file 7.** Phylogenetic Reconstruction II—Maximum Likelihood fits of 24 different nucleotide substitution models. Summarizing table for the models with the lowest BIC scores (Bayesian Information Criterion) that are considered to describe the substitution pattern the best.

**Additional file 8.** Phylogenetic Reconstruction III—Neighbor-Joining Tree. Neighbor-Joining (NJ) tree illustrating distances and phylogenetic relationships among porcine haplotypes of the 637 bp-alignment of the mitochondrial control region of 42 domestic, wild and prehistoric domestic pigs.

**Additional file 9.** Supplementary references. Publications being cited in Additional files 1–9.

## Abbreviations

UNESCO: United Nations Educational, Scientific and Cultural Organization
BC: before Christ
MASL: meters above sea level
mt: mitochondrial
CR: control region
NHMW: Natural History Museum Vienna
EDTA: ethylene diamine tetraacetic acid
PCR: polymerase chain reaction
bp: base pairs
NJ: Neighbor-Joining
ML: Maximum-Likelihood
BI: Bayesian inference
MEGA: molecular evolutionary genetics analysis
numts: nuclear DNA sequences of mitochondrial origin
MCR1: melanocortin receptor 1

## Footnotes

1 Molecular BioProducts (San Diego, CA).

2 Qiagen (Hilden, Germany).

3 Retsch GmbH (Haan, Germany).

4 Carl Roth (Karlsruhe, Germany).

5 Eppendorf AG (Hamburg, Germany).

6 GEN-IAL (Troisdorf, Germany).

7 Thermo Fisher Scientific (Waltham, MA).

8 Microsynth AG (Balgach, Switzerland).

9 LGC Genomics (Berlin, Germany).
