# Supplementary material for: Bronze Age meat industry: ancient mitochondrial DNA analyses of pig bones from the prehistoric salt mines of Hallstatt (Austria)
Source: BMC Res Notes. 2018 Apr 13;11:243. doi: 10.1186/s13104-018-3340-7 (PMC5899323; doi:10.1186/s13104-018-3340-7)
Supplement: Supplementary file 1 — Additional file 1. Ancient DNA extraction and Mitochondrial control region PCR. Detailed protocols for Ancient DNA extraction and PCR of mitochondrial control region. [file 13104_2018_3340_MOESM1_ESM.pdf]

ADDITIONAL FILE 1: Material and Methods

Ancient DNA extraction and Mitochondrial control region PCR.

Ancient DNA extraction

All DNA extractions were performed in a clean room by obeying all standard routines for working with aDNA, e.g., regularly overnight UV irradiation of the lab, treatment of work surfaces with *DNA-Away*[1] (for manufacturers see below) and 3% sodium hypochlorite, extra UV irradiation of tubes and other equipment (Cooper and Poinar 2000; Haring et al. 2015). The use of disposable coveralls, masks, hairnets and gloves as well as sterile RNA/DNA free manufactured plastic and filter tips was mandatory throughout the entire procedure. Cleaning and decontamination of grinding jars and balls was performed with *DNA-Away*[1] after an ultrasonic bath followed by subsequent UV radiation. All post-PCR work was performed in a separate laboratory and extraction controls (buffers without sample) were performed to screen for contaminated reagents. For each specimen, at least two independent DNA extractions and sequencing reads were performed.

Prior to the DNA extraction, the surface of each tooth was decontaminated as described in Watt (2005) by treatment with 3% sodium hypochlorite for 10 min followed by rinsing in nuclease free water[2] for 10 min. After drying the teeth were cautiously crushed in a sterile plastic bag using a small hammer and the small pieces were finally pulverized with a Retsch MM400 grinding mill using a 10 ml zirconium oxide grinding jar and a 12 mm zirconium oxide grinding ball[3]. To avoid extreme heat generation, only a small amount of pieces (max. 300 mm^3) was grinded separately for 1 minute at 30 Hz. Preferentially, compact pieces were taken and the porous ones avoided. The powder was transferred to a sterile DNA free 5 ml Safelock tube[2] until 1 g was reached. Decalcification of the tooth powder was done using *Decalcifier soft*[4] (containing 25% EDTA) in a 5 ml Safelock tube (4.5 ml per 1 g tooth powder). Tubes were sealed with parafilm and incubated with rotation overnight at 4°C. After 10 min centrifugation at 4,000 rpm (Eppendorf Centrifuge 5430[5], Eppendorf AG, Hamburg, Germany) the supernatant was discarded and replaced by fresh (4.5 ml) decalcifying solution. The tooth powder was dissolved by stirring with a sterile spatula, shortly vortexed

and again incubated with rotation overnight at 4°C. This procedure was repeated three times. After decalcification, the powder was washed to remove all remains of EDTA by adding 4.5 ml nuclease free water to each sample and after vortexing the tubes were centrifuged 10 min at 4,000 rpm. The supernatant was discarded and the washing step was repeated three times.

DNA was extracted with the *Gen-ial All Tissue Kit*[6] according to the manufacturer's instructions for DNA extraction from bone and teeth. Each sample (rinsed powder in 5 ml tube) 1 ml lysis buffer 1, 100 µl lysis buffer 2, 50 µl proteinase K and 25 µl 1 M dithiotreitol were added. The tube was sealed with parafilm and incubated with rotation at 56°C for 3-4 days until the powder was completely resolved. After 15 min centrifugation at 7,830 rpm the clear supernatant was transferred to 2 ml tubes and 0.75 volume of lysis buffer 3 was added to each tube. After short vortexing the tubes were incubated at -20°C for 5 min, followed by centrifugation for 10 min at 13,000 rpm. Next, the supernatant was transferred to a new 2 ml tube and the DNA was precipitated by adding 640 µl isopropanol and 30 min incubation at 4°C. After 15 min centrifugation at 13,000 rpm, the supernatant was discarded and the pellet was washed with 300 µl cold 70% ethanol and centrifuged for 5 min at 13,000 rpm. Next, the ethanol was discarded and the pellet dried for several hours. Finally, the DNA was dissolved in 30 µl nuclease free water[2] and the concentrations were measured with the Eppendorf BioPhotometer D30[5] (µCuvette G1.0). To avoid degradation of DNA by repeated freezing/thawing, the DNA solutions were immediately aliquoted (5-10 µl) and stored at 4°C (short term) or at -20 °C for long term.

PCR of mitochondrial control region

A 721-basepair-long section of the mitochondrial (mt) control region (CR) was amplified using three PCR primer pairs that produce overlapping amplicons, ranging from 343 bp to 401 bp in length (Online Resources 2 and 3). The PCR was performed with Amplitaq Gold® 360 DNA-Polymerase[7] in 50 µl with 1x AmpliTaq Gold® 360 Buffer, 0.5 µM of each primer[8], 0.2 mM of each dNTP[2] and 2 µl DNA solution. PCR reactions were run on a Mastercycler Nexus[5]. Details of primer combinations are given in Table 2. The thermal cycling conditions

for the fragment A (401 bp) involved the use of a touch-down protocol to account for the distant melting temperatures (more than 5°C) of these two primers. An initial activation step of 95°C for 10 minutes was followed by two cycles of denaturation at 95°C for 30 seconds, 30 seconds primer annealing at 67°C and 40 seconds primer extension at 72°C. The following 45 cycles were run at 95°C denaturation for 30 seconds, 58°C annealing temperature for 30 seconds and 40 seconds primer extension at 72°C, completed by a final extension step of 7 minutes at 72°C. PCR cycling conditions for fragment B (343 bp) and C (392 bp) comprised a conventional PCR protocol: initial activation at 95°C for 10 minutes; 45 cycles of 95°C for 30 seconds, primer annealing at 52°C (fragment B) and 50°C (fragment C) for 30 seconds and primer extension at 72°C for 40 seconds, completed by a final extension step of 7 minutes at 72°C. Unsuccessful PCR reactions were repeated with varying amounts of template DNA. Control PCR reactions were performed to screen for contaminated reagents: extraction control (buffers without sample) and non-template control with nuclease-free water instead of template. Finally, PCR products were purified with the QIAquick PCR Purification Kit[2] and sequences using the PCR primers at Microsynth AG[8] and LGC Genomics[9].

List of manufacturers

[1]Molecular BioProducts (San Diego, CA)

[2]Qiagen (Hilden, Germany)

[3]Retsch GmbH (Haan, Germany)

[4]Carl Roth (Karlsruhe, Germany)

[5]Eppendorf AG (Hamburg, Germany)

[6]GEN-IAL (Troisdorf, Germany)

[7]Thermo Fisher Scientific (Waltham, MA)

[8]Microsynth AG (Balgach, Switzerland)

[9]LGC Genomics (Berlin, Germany)
