# Supplementary material for: Bronze Age meat industry: ancient mitochondrial DNA analyses of pig bones from the prehistoric salt mines of Hallstatt (Austria)
Source: BMC Res Notes. 2018 Apr 13;11:243. doi: 10.1186/s13104-018-3340-7 (PMC5899323; doi:10.1186/s13104-018-3340-7)
Supplement: Supplementary file 2 — Additional file 2. Map of the porcine mitochondrial DNA. Localisation of the 721 bp long control region (CR) fragment in the reference mitochondrial genome. [file 13104_2018_3340_MOESM2_ESM.pdf]

ADDITIONAL FILE 2: Material and Methods

Localisation of the 721 bp long control region (CR) fragment in the reference mitochondrial genome AF034253 (Lin et al. 1999).

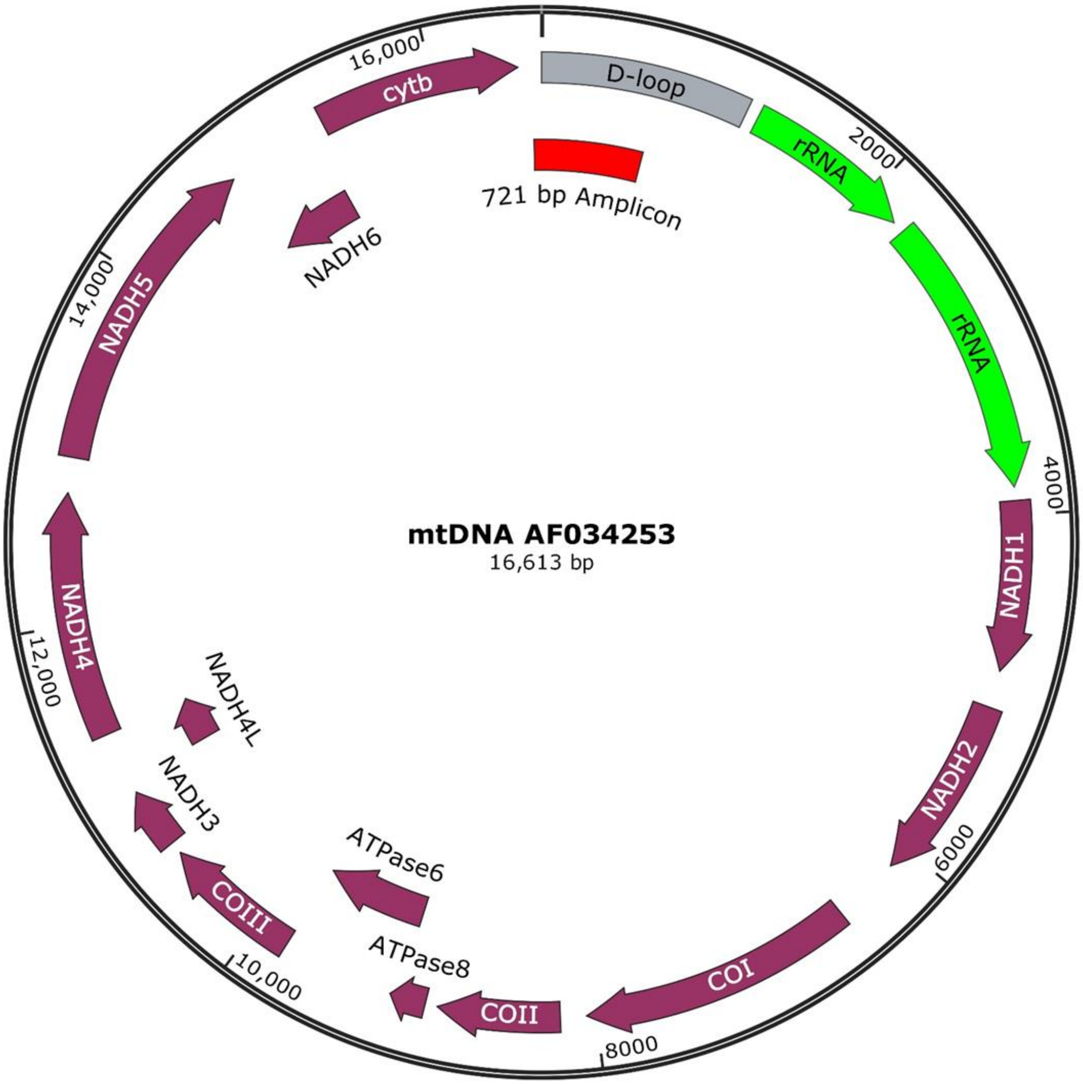

Created with SnapGene® version 3.3 (GSL Biotech, Chicago, IL, USA). The 721-bp-long control region fragment of the mtDNA is indicated by the red box.
