# Supplementary material for: Bronze Age meat industry: ancient mitochondrial DNA analyses of pig bones from the prehistoric salt mines of Hallstatt (Austria)
Source: BMC Res Notes. 2018 Apr 13;11:243. doi: 10.1186/s13104-018-3340-7 (PMC5899323; doi:10.1186/s13104-018-3340-7)
Supplement: Supplementary file 3 — Additional file 3. PCR strategy for the 721-bp-long section of the mitochondrial Control Region (CR). The CR sequences were inferred using three PCR primer pairs that allow amplification of overlapping amplicons, ranging from 343 bp to 401 bp in length. [file 13104_2018_3340_MOESM3_ESM.pdf]

ADDITIONAL FILE 3: Material and Methods

PCR strategy for the 721-bp-long section of the mitochondrial Control Region (CR).

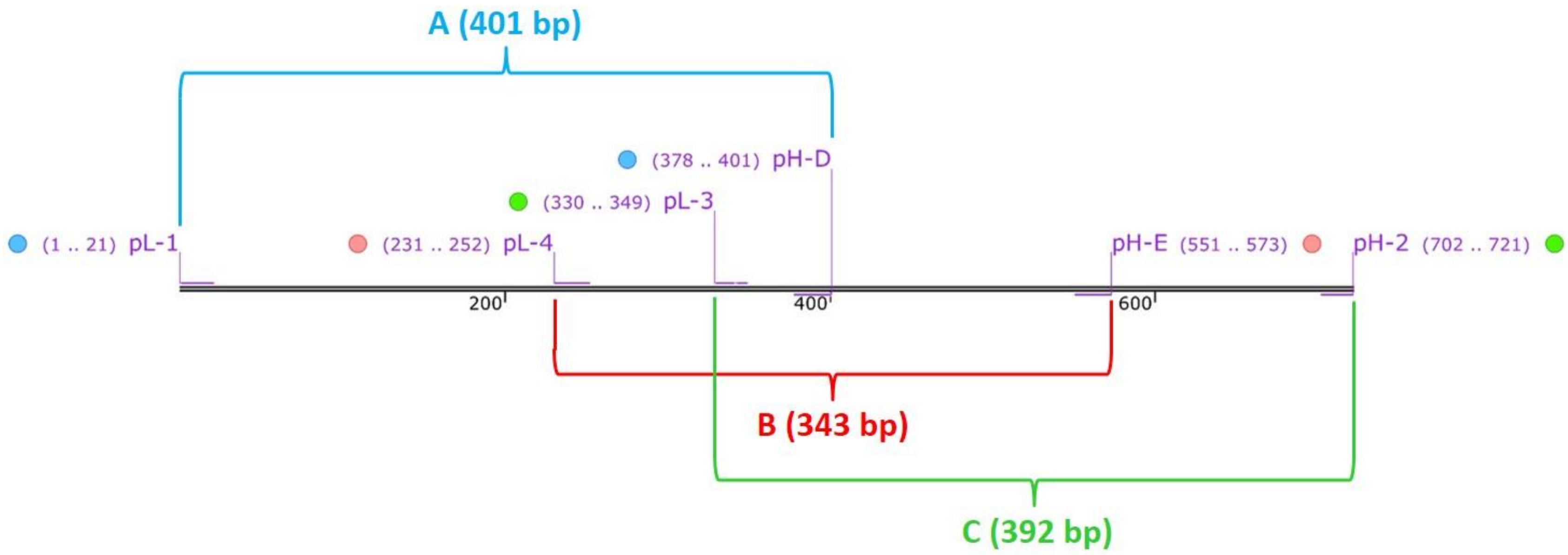

Created with SnapGene® version 3.3 (GSL Biotech, Chicago, IL, USA). The 721-bp-long control region section is located from position nt -48 (16,566) to nt +673 of the mitochondrial genome sequence (Acc No AF034253, Lin et al. 1999). The CR sequences were inferred using three PCR primer pairs that allow amplification of overlapping amplicons, ranging from 343 bp to 401 bp in length. Primer names and binding sites are indicated (see also Table in Additional File 4).
