# Supplementary material for: Bronze Age meat industry: ancient mitochondrial DNA analyses of pig bones from the prehistoric salt mines of Hallstatt (Austria)
Source: BMC Res Notes. 2018 Apr 13;11:243. doi: 10.1186/s13104-018-3340-7 (PMC5899323; doi:10.1186/s13104-018-3340-7)
Supplement: Supplementary file 4 — Additional file 4. PCR strategy for the 721-bp-long section of the mitochondrial Control Region (CR). Primers used to amplify three sections (A, B, C) of the mitochondrial control region. [file 13104_2018_3340_MOESM4_ESM.pdf]

ADDITIONAL FILE 4: Material and Methods

Primers used to amplify three sections (A, B, C) of the mitochondrial control region.

Fragment length	Primer	Sequence (5' → 3')	Binding sites[a]	Orientation	Ref.	T_A
A – 401 bp	pL-1	CTCCGCCATCAGCACCCAAAG	16,566-16,586	fwd	Larson et al. (2005)	67/58°C
	pH-D	AGCGGGTTGCTGGTTTCACGCGGC	330-353	rev	This study	67/58°C
B – 343 bp	pL-4	GCTAGTCCCCATGCATATAAGC	183-204	fwd	This study	52°C
	pH-E	GTGTGAGCATGGGCTGATTAGTC	505-525	rev	This study	52°C
C – 392 bp	pL-3	CAGTCAACATGCGTATCACC	282-301	fwd	Watanobe et al. (2001)	50°C
	pH-2	GCACCTTGTTTGGATTGTCG	654-673	rev	Watanobe et al. (2001)	50°C

[a]Mitochondrial genome positions are given according to Accession number AF034253 (Lin et al. 1999). Note: L = light strand; H = heavy strand; fwd = forward; rev = reverse; Ref = reference; T_A = annealing temperature for PCR. Primer combination pL1/pH-2 was used to amplify the 721-bp long fragment.
