# Supplementary material for: Bronze Age meat industry: ancient mitochondrial DNA analyses of pig bones from the prehistoric salt mines of Hallstatt (Austria)
Source: BMC Res Notes. 2018 Apr 13;11:243. doi: 10.1186/s13104-018-3340-7 (PMC5899323; doi:10.1186/s13104-018-3340-7)
Supplement: Supplementary file 5 — Additional file 5. Detailed sequencing outcome for the 10 prehistoric porcine teeth specimens. For each specimen, DNA concentration and the number of obtained sequence reads for fragments A, B and C, are given. [file 13104_2018_3340_MOESM5_ESM.pdf]

ADDITIONAL FILE 5: Results and Discussion

Detailed sequencing outcome for the 10 prehistoric porcine teeth specimens.

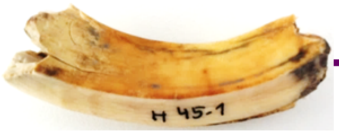

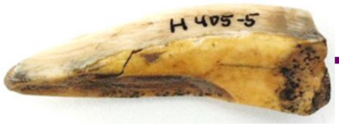

Specimen	[DNA]	Frag A (401bp)	Frag B (343bp)	Frag C (392bp)
**H45-1**	**7.9 ng/µl**	**2**	**2**	**2**
H94-2A	6.0 ng/µl	0	0	0
**H136-3**	**6.3 ng/µl**	0	**4**	0
**HoN-4**	**16.9 ng/µl**	**4**	**4**	**2**
**H405-5**	**14.9 ng/µl**	**2**	**6**	**4**
**H124-6**	**13.6 ng/µl**	**2**	**2**	**2**
H188-7	20.6 ng/µl	0	0	0
**H288-7**	**10.1 ng/µl**	**2**	**4**	**2**
**H51-1**	**8.2 ng/µl**	**2**	**2**	**2**
**H117-21**	**4.6 ng/µl**	**2**	**2**	**2**

Colour codes of fragments A, B, C correspond to schematic drawing in Additional File 3. For each fragment, the number of obtained sequence reads are given. White colour indicates that the expected PCR product could not be amplified.
