# Supplementary material for: Bronze Age meat industry: ancient mitochondrial DNA analyses of pig bones from the prehistoric salt mines of Hallstatt (Austria)
Source: BMC Res Notes. 2018 Apr 13;11:243. doi: 10.1186/s13104-018-3340-7 (PMC5899323; doi:10.1186/s13104-018-3340-7)
Supplement: Supplementary file 6 — Additional file 6. Phylogenetic Reconstruction I—estimates of evolutionary divergence between sequences. Distance matrix shows the number of base differences per site (p distances; Nei and Kumar 2000) between 42 DNA sequences (length of alignment 637 bp). [file 13104_2018_3340_MOESM6_ESM.pdf]

ADDITIONAL FILE 6: Results and Discussion

Phylogenetic Reconstruction I – Estimates of evolutionary divergence between sequences.

No	Taxon	1	2	3	4	5	6	7	8	9	10	11	12	13	14	15	16	17	18	19	20	21	22	23	24	25	26	27	28	29	30	31	32	33	34	35	36	37	38	39	40	41	42
1	Sus_cebifrons	0.000	0.025	0.027	0.039	0.039	0.043	0.050	0.047	0.047	0.052	0.047	0.044	0.049	0.049	0.036	0.041	0.042	0.049	0.049	0.049	0.047	0.046	0.046	0.047	0.044	0.047	0.038	0.042	0.046	0.039	0.043	0.041	0.041	0.039	0.046	0.049	0.041	0.049	0.057	0.046	0.052	0.047
2	Sus_barbatus			0.011	0.024	0.030	0.030	0.035	0.035	0.031	0.036	0.035	0.035	0.030	0.033	0.027	0.028	0.033	0.036	0.033	0.036	0.028	0.036	0.033	0.035	0.028	0.028	0.025	0.027	0.030	0.027	0.030	0.028	0.028	0.030	0.033	0.033	0.031	0.033	0.041	0.033	0.036	0.035
3	Sus_verrucosus				0.028	0.028	0.028	0.039	0.039	0.036	0.041	0.036	0.039	0.038	0.038	0.025	0.027	0.038	0.038	0.038	0.038	0.036	0.038	0.038	0.039	0.033	0.036	0.024	0.028	0.031	0.028	0.028	0.027	0.027	0.028	0.031	0.038	0.030	0.038	0.046	0.038	0.041	0.039
4	Sus_scrofa_papuensis					0.019	0.016	0.027	0.027	0.024	0.028	0.027	0.024	0.022	0.025	0.016	0.017	0.022	0.028	0.025	0.022	0.024	0.028	0.022	0.024	0.019	0.020	0.014	0.022	0.019	0.016	0.019	0.017	0.017	0.019	0.022	0.025	0.017	0.025	0.033	0.022	0.028	0.024
5	Sus_scrofa_taiwanensis						0.013	0.027	0.027	0.024	0.028	0.024	0.024	0.025	0.025	0.006	0.011	0.022	0.025	0.025	0.022	0.024	0.025	0.022	0.024	0.025	0.024	0.005	0.016	0.009	0.013	0.009	0.008	0.011	0.013	0.013	0.025	0.014	0.025	0.036	0.022	0.028	0.024
6	Sus_scrofa_andamensis							0.024	0.024	0.020	0.025	0.020	0.020	0.022	0.022	0.009	0.002	0.019	0.022	0.022	0.019	0.020	0.022	0.019	0.020	0.022	0.020	0.008	0.019	0.009	0.009	0.006	0.005	0.005	0.006	0.009	0.022	0.008	0.022	0.033	0.019	0.025	0.020
7	Large_White(EU)								0.006	0.006	0.005	0.006	0.006	0.008	0.005	0.024	0.025	0.008	0.008	0.005	0.008	0.006	0.008	0.005	0.003	0.020	0.016	0.022	0.024	0.030	0.027	0.027	0.025	0.025	0.027	0.027	0.005	0.019	0.005	0.019	0.005	0.008	0.006
8	Large_White(F)									0.003	0.005	0.003	0.003	0.005	0.002	0.020	0.025	0.005	0.005	0.002	0.008	0.006	0.002	0.005	0.006	0.020	0.019	0.022	0.024	0.030	0.024	0.027	0.025	0.025	0.024	0.030	0.002	0.016	0.002	0.016	0.005	0.005	0.006
9	Linderodssvin(S)										0.005	0.003	0.006	0.002	0.002	0.020	0.022	0.008	0.005	0.002	0.008	0.003	0.005	0.005	0.006	0.017	0.016	0.019	0.024	0.027	0.024	0.024	0.022	0.022	0.024	0.027	0.002	0.019	0.002	0.013	0.005	0.005	0.006
10	Pietrain(D)											0.005	0.008	0.006	0.003	0.025	0.027	0.009	0.006	0.003	0.009	0.008	0.006	0.006	0.005	0.022	0.017	0.024	0.025	0.031	0.028	0.028	0.027	0.027	0.028	0.028	0.003	0.020	0.003	0.017	0.006	0.006	0.008
11	Mangalica(H)												0.006	0.005	0.002	0.020	0.022	0.008	0.002	0.002	0.008	0.006	0.002	0.005	0.006	0.020	0.019	0.019	0.027	0.027	0.024	0.024	0.022	0.022	0.024	0.027	0.002	0.016	0.002	0.016	0.005	0.005	0.006
12	Landrace-1(FI)													0.008	0.005	0.017	0.022	0.002	0.008	0.005	0.005	0.006	0.005	0.002	0.003	0.017	0.016	0.019	0.024	0.027	0.020	0.024	0.022	0.022	0.020	0.027	0.005	0.013	0.005	0.019	0.002	0.008	0.003
13	Landrace-2(N)														0.003	0.022	0.024	0.009	0.006	0.003	0.009	0.005	0.006	0.006	0.008	0.016	0.014	0.020	0.025	0.025	0.022	0.025	0.024	0.024	0.025	0.028	0.003	0.020	0.003	0.014	0.006	0.006	0.008
14	Duroc(UK)															0.022	0.024	0.006	0.003	0.000	0.006	0.005	0.003	0.003	0.005	0.019	0.017	0.020	0.025	0.028	0.025	0.025	0.024	0.024	0.025	0.028	0.000	0.017	0.000	0.014	0.003	0.003	0.005
15	Saddleback(D)																0.008	0.016	0.022	0.022	0.019	0.020	0.019	0.019	0.020	0.022	0.020	0.002	0.013	0.009	0.006	0.006	0.005	0.008	0.006	0.013	0.022	0.008	0.022	0.033	0.019	0.025	0.020
16	Creole(F)																	0.020	0.024	0.024	0.020	0.022	0.024	0.020	0.022	0.024	0.022	0.006	0.017	0.008	0.008	0.005	0.003	0.003	0.005	0.008	0.024	0.009	0.024	0.035	0.020	0.027	0.022
17	Wild_boar(A)																		0.009	0.006	0.006	0.008	0.006	0.003	0.005	0.016	0.014	0.017	0.022	0.025	0.019	0.022	0.020	0.020	0.019	0.025	0.006	0.011	0.006	0.020	0.003	0.009	0.005
18	Wild_boar(D)																			0.003	0.009	0.008	0.003	0.006	0.008	0.022	0.020	0.020	0.028	0.028	0.025	0.025	0.024	0.024	0.025	0.028	0.003	0.017	0.003	0.017	0.006	0.006	0.008
19	Wild_boar-1(F)																				0.006	0.005	0.003	0.003	0.005	0.019	0.017	0.020	0.025	0.028	0.025	0.025	0.024	0.024	0.025	0.028	0.000	0.017	0.000	0.014	0.003	0.003	0.005
20	Wild_boar-2(F)																					0.008	0.009	0.003	0.005	0.016	0.017	0.017	0.025	0.025	0.022	0.022	0.020	0.020	0.022	0.025	0.006	0.014	0.006	0.020	0.003	0.009	0.005
21	Wild_boar(B)																						0.008	0.005	0.006	0.017	0.016	0.019	0.024	0.027	0.024	0.024	0.022	0.022	0.024	0.027	0.005	0.019	0.005	0.016	0.005	0.008	0.006
22	Wild_boar(E)																							0.006	0.008	0.022	0.020	0.020	0.025	0.028	0.022	0.025	0.024	0.024	0.022	0.028	0.003	0.014	0.003	0.017	0.006	0.006	0.008
23	Wild_boar(N)																								0.002	0.016	0.014	0.017	0.025	0.025	0.022	0.022	0.020	0.020	0.022	0.025	0.003	0.014	0.003	0.017	0.000	0.006	0.002
24	Wild_boar(MK)																									0.017	0.013	0.019	0.024	0.027	0.024	0.024	0.022	0.022	0.024	0.024	0.005	0.016	0.005	0.019	0.002	0.008	0.003
25	Wild_boar(AM)																										0.017	0.020	0.028	0.022	0.022	0.025	0.024	0.024	0.025	0.025	0.019	0.020	0.019	0.030	0.016	0.022	0.017
26	Wild_boar-1(IR)																											0.019	0.020	0.024	0.020	0.024	0.022	0.019	0.020	0.024	0.017	0.019	0.017	0.028	0.014	0.020	0.016
27	Wild_boar-2(IR)																												0.014	0.008	0.008	0.005	0.003	0.006	0.008	0.011	0.020	0.009	0.020	0.032	0.017	0.024	0.019
28	Wild_boar(IN)																													0.019	0.016	0.019	0.017	0.017	0.016	0.016	0.025	0.017	0.025	0.036	0.025	0.028	0.027
29	Wild_boar(ID)																														0.009	0.006	0.005	0.008	0.009	0.006	0.028	0.014	0.028	0.039	0.025	0.031	0.027
30	Wild_boar-1(CN)																															0.009	0.008	0.008	0.006	0.013	0.025	0.008	0.025	0.036	0.022	0.028	0.024
31	Wild_boar-2(CN)																																0.002	0.005	0.006	0.009	0.025	0.011	0.025	0.036	0.022	0.028	0.024
32	Zang(CN)																																	0.003	0.005	0.008	0.024	0.009	0.024	0.035	0.020	0.027	0.022
33	Bamei(CN)																																		0.002	0.008	0.024	0.009	0.024	0.035	0.020	0.027	0.022
34	Huzhu(CN)																																			0.009	0.025	0.008	0.025	0.036	0.022	0.028	0.024
35	Meishan(CN)																																				0.028	0.014	0.028	0.039	0.025	0.031	0.027
36	H45-1(A)																																					0.017	0.000	0.014	0.003	0.003	0.005
37	HoN-4(A)																																						0.017	0.032	0.014	0.020	0.016
38	H405-5(A)																																							0.014	0.003	0.003	0.005
39	H124-6(A)																																								0.017	0.017	0.019
40	H288-7(A)																																									0.006	0.002
41	H51-1(A)																																										0.008
42	H117-21(A)																																										0.000

Note. The number of base differences per site (*p* distances; Nei and Kumar 2000) between 42 DNA sequences (length of alignment 637 bp). All ambiguous positions were removed for each sequence pair. Sequences of prehistoric domestic pigs obtained in this study are given in red.
