# Supplementary material for: Bronze Age meat industry: ancient mitochondrial DNA analyses of pig bones from the prehistoric salt mines of Hallstatt (Austria)
Source: BMC Res Notes. 2018 Apr 13;11:243. doi: 10.1186/s13104-018-3340-7 (PMC5899323; doi:10.1186/s13104-018-3340-7)
Supplement: Supplementary file 7 — Additional file 7. Phylogenetic Reconstruction II—Maximum Likelihood fits of 24 different nucleotide substitution models. Summarizing table for the models with the lowest BIC scores (Bayesian Information Criterion) that are considered to describe the substitution pattern the best. [file 13104_2018_3340_MOESM7_ESM.pdf]

ADDITIONAL FILE 7: Results and Discussion

Phylogenetic Reconstruction II – Maximum Likelihood fits of 24 different nucleotide substitution models.

Model	#Param	BIC	AICc	lnL	Invariant	Gamma	R	Freq A	Freq T	Freq C	Freq G	A=>T	A=>C	A=>G	T=>A	T=>C	T=>G	C=>A	C=>T	C=>G	G=>A	G=>T	G=>C
T92+G+I	85	3981.791587	3285.863649	-1557.657549	0.748087222	0.57668354	27.20209873	0.303519336	0.303519336	0.196480664	0.196480664	0.01	0	0.19	0.01	0.19	0	0.01	0.29	0	0.29	0.01	0
HKY+G	86	3995.221968	3291.113117	-1559.275819	n/a	0.06976849	25.24604094	0.327324407	0.279639464	0.246615304	0.146346024	0.01	0	0.14	0.01	0.24	0	0.01	0.27	0	0.32	0.01	0
T92+G	84	3999.596192	3311.849318	-1571.656772	n/a	0.059824474	25.05396569	0.303519336	0.303519336	0.196480664	0.196480664	0.01	0	0.19	0.01	0.19	0	0.01	0.29	0	0.29	0.01	0
K2+G+I	84	3999.921146	3312.174272	-1571.819249	0.753551181	0.619719655	26.53314169	0.25	0.25	0.25	0.25	0	0	0.24	0	0.24	0	0	0.24	0	0.24	0	0
TN93+G	87	4005.15923	3292.869617	-1559.147529	n/a	0.075868543	25.28335924	0.327324407	0.279639464	0.246615304	0.146346024	0.01	0	0.13	0.01	0.25	0	0.01	0.29	0	0.29	0.01	0
K2+G	83	4017.049345	3337.483686	-1585.480269	n/a	0.05	25.06068037	0.25	0.25	0.25	0.25	0	0	0.24	0	0.24	0	0	0.24	0	0.24	0	0
GTR+G+I	91	4017.230761	3272.219613	-1544.795613	0.736718806	0.593332722	26.5359398	0.327324407	0.279639464	0.246615304	0.146346024	0.01	0	0.12	0.01	0.27	0	0	0.3	0	0.27	0	0.01
TN93+G+I	88	4030.796946	3310.326722	-1566.869466	0.753500188	0.530021236	29.1894686	0.327324407	0.279639464	0.246615304	0.146346024	0	0	0.13	0.01	0.26	0	0.01	0.29	0	0.29	0	0
GTR+G	90	4037.469823	3300.638831	-1560.012064	n/a	0.068862623	18.44531724	0.327324407	0.279639464	0.246615304	0.146346024	0.01	0.01	0.13	0.01	0.24	0	0.01	0.28	0	0.3	0.01	0.01
T92+I	84	4073.335256	3385.588382	-1608.526304	0.445054945	n/a	23.857068	0.303519336	0.303519336	0.196480664	0.196480664	0.01	0	0.19	0.01	0.19	0	0.01	0.29	0	0.29	0.01	0
K2+I	83	4094.678529	3415.11287	-1624.294861	0.445054945	n/a	23.75278569	0.25	0.25	0.25	0.25	0.01	0.01	0.24	0.01	0.24	0.01	0.01	0.24	0.01	0.24	0.01	0.01
HKY+I	86	4103.471455	3399.362604	-1613.400562	0.445054945	n/a	25.03461045	0.327324407	0.279639464	0.246615304	0.146346024	0.01	0	0.14	0.01	0.24	0	0.01	0.27	0	0.32	0.01	0
GTR+I	90	4108.905243	3372.074251	-1595.729774	0.445054945	n/a	15.79848338	0.327324407	0.279639464	0.246615304	0.146346024	0.01	0.01	0.13	0.01	0.25	0	0.01	0.29	0	0.28	0.01	0.01
TN93+I	87	4112.168168	3399.878556	-1612.651998	0.445054945	n/a	25.13059829	0.327324407	0.279639464	0.246615304	0.146346024	0.01	0	0.12	0.01	0.27	0	0.01	0.3	0	0.27	0.01	0
T92	83	4126.28499	3446.719331	-1640.098091	n/a	n/a	23.51735827	0.303519336	0.303519336	0.196480664	0.196480664	0.01	0	0.19	0.01	0.19	0	0.01	0.29	0	0.29	0.01	0
HKY	85	4131.789986	3435.862048	-1632.656748	n/a	n/a	24.11727244	0.327324407	0.279639464	0.246615304	0.146346024	0.01	0	0.14	0.01	0.24	0	0.01	0.27	0	0.32	0.01	0
TN93	86	4140.45487	3436.346019	-1631.89227	n/a	n/a	24.11891838	0.327324407	0.279639464	0.246615304	0.146346024	0.01	0	0.12	0.01	0.26	0	0.01	0.3	0	0.28	0.01	0
K2	82	4141.926892	3470.542599	-1653.015963	n/a	n/a	23.48255176	0.25	0.25	0.25	0.25	0.01	0.01	0.24	0.01	0.24	0.01	0.01	0.24	0.01	0.24	0.01	0.01
GTR	89	4154.850589	3426.199906	-1623.799368	n/a	n/a	18.86799028	0.327324407	0.279639464	0.246615304	0.146346024	0.01	0	0.13	0.01	0.26	0	0	0.29	0	0.28	0.01	0.01
JC+G+I	83	4214.673834	3535.108175	-1684.292513	0.748946699	0.674699766	0.5	0.25	0.25	0.25	0.25	0.08	0.08	0.08	0.08	0.08	0.08	0.08	0.08	0.08	0.08	0.08	0.08
JC+G	82	4228.340948	3556.956655	-1696.222991	n/a	0.05	0.5	0.25	0.25	0.25	0.25	0.08	0.08	0.08	0.08	0.08	0.08	0.08	0.08	0.08	0.08	0.08	0.08
JC+I	82	4303.101313	3631.71702	-1733.603173	0.445054945	n/a	0.5	0.25	0.25	0.25	0.25	0.08	0.08	0.08	0.08	0.08	0.08	0.08	0.08	0.08	0.08	0.08	0.08
JC	81	4349.714699	3686.511923	-1762.006787	n/a	n/a	0.5	0.25	0.25	0.25	0.25	0.08	0.08	0.08	0.08	0.08	0.08	0.08	0.08	0.08	0.08	0.08	0.08
HKY+G+I	87	26757.69491	26045.4053	-12935.41537	0.73742396	0.62344565	25.37299878	0.327324407	0.279639464	0.246615304	0.146346024	0	0	0.14	0.01	0.24	0	0.01	0.27	0	0.32	0	0

Note: Models with the lowest BIC scores (Bayesian Information Criterion) are considered to describe the substitution pattern the best. For each model, AICc value (Akaike Information Criterion, corrected), Maximum Likelihood value (lnL), and the number of parameters (including branch lengths) are also presented (Nei and Kumar 2000). Non-uniformity of evolutionary rates among sites may be modelled by using a discrete Gamma distribution (+G) with 5 rate categories and by assuming that a certain fraction of sites are evolutionarily invariable (+I). Whenever applicable, estimates of gamma shape parameter and/or the estimated fraction of invariant sites are shown. Assumed or estimated values of transition/transversion bias (R) are shown for each model, as well. They are followed by nucleotide frequencies (f) and rates of base substitutions (r) for each nucleotide pair. Relative values of instantaneous r should be considered when evaluating them. For simplicity, sum of r values is made equal to 1 for each model. For estimating ML values, a tree topology was automatically computed. The analysis involved 42 nucleotide sequences. There were a total of 637 positions in the final dataset. Evolutionary analyses were conducted in MEGA7 (ver7.0.14, Kumar et al. 2016). Abbreviations: GTR: General Time Reversible; HKY: Hasegawa-Kishino-Yano; TN93: Tamura-Nei; T92: Tamura 3-parameter; K2: Kimura 2-parameter; JC: Jukes-Cantor.
