# Supplementary material for: Bronze Age meat industry: ancient mitochondrial DNA analyses of pig bones from the prehistoric salt mines of Hallstatt (Austria)
Source: BMC Res Notes. 2018 Apr 13;11:243. doi: 10.1186/s13104-018-3340-7 (PMC5899323; doi:10.1186/s13104-018-3340-7)
Supplement: Supplementary file 8 — Additional file 8. Phylogenetic Reconstruction III—Neighbor-Joining Tree. Neighbor-Joining (NJ) tree illustrating distances and phylogenetic relationships among porcine haplotypes of the 637 bp-alignment of the mitochondrial control region of 42 domestic, wild and prehistoric domestic pigs. [file 13104_2018_3340_MOESM8_ESM.pdf]

ADDITIONAL FILE 8: Results and Discussion

Phylogenetic Reconstruction III – Neighbor-Joining Tree.

Neighbor-Joining (NJ) tree illustrating distances and phylogenetic relationships among porcine haplotypes of the 637 bp-alignment of the mitochondrial control region of 42 domestic, wild and prehistoric domestic pigs (in red). Bootstrap values (>50%) and posterior probability values (>0.97) of the BI analysis are given next to the nodes: NJ (left), ML (middle), BI (right). The tree is drawn to scale, with branch lengths reflecting *p* distances (scale bar = 0.005 substitutions per site). For more details on taxa and samples see Table 1. The tree was rooted with three related Suinae: *Sus barbatus* (Bornean bearded pig), *Sus cebifrons* (Visayan warty pig) and *Sus verrucosus* (Java warty pig).

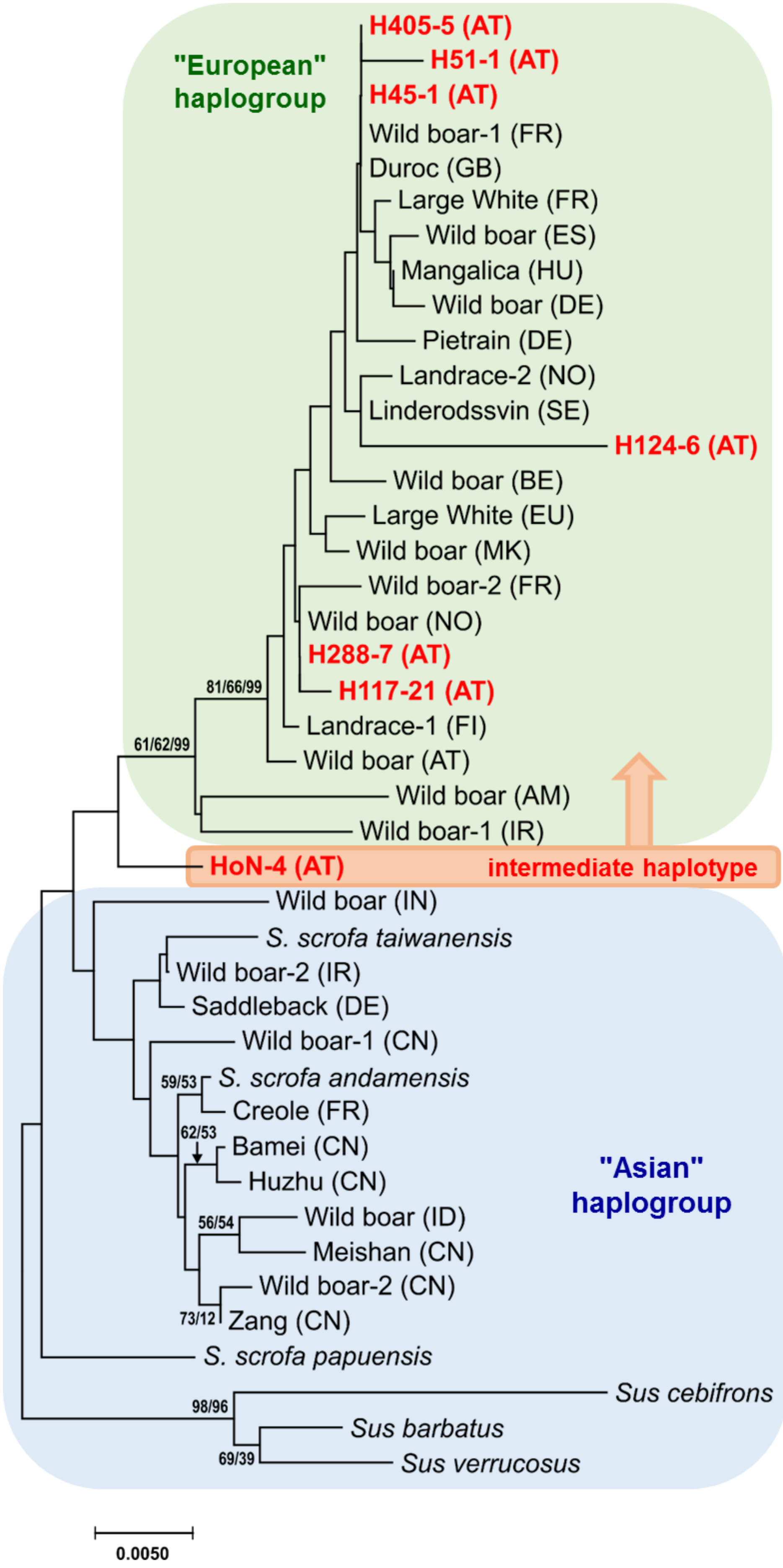
"European" haplogroup
H405-5 (AT)
H51-1 (AT)
H45-1 (AT)
Wild boar-1 (FR)
Duroc (GB)
Large White (FR)
Wild boar (ES)
Mangalica (HU)
Wild boar (DE)
Pietrain (DE)
Landrace-2 (NO)
Linderodssvin (SE)
H124-6 (AT)
Wild boar (BE)
Large White (EU)
Wild boar (MK)
Wild boar-2 (FR)
Wild boar (NO)
H288-7 (AT)
81/66/99
H117-21 (AT)
Landrace-1 (FI)
61/62/99
Wild boar (AT)
Wild boar (AM)
Wild boar-1 (IR)
HoN-4 (AT)
intermediate haplotype
Wild boar (IN)
S. scrofa taiwanensis
Wild boar-2 (IR)
Saddleback (DE)
Wild boar-1 (CN)
59/53
S. scrofa andamensis
Creole (FR)
62/53
Bamei (CN)
Huzhu (CN)
"Asian" haplogroup
56/54
Wild boar (ID)
Meishan (CN)
Wild boar-2 (CN)
73/12
Zang (CN)
S. scrofa papuensis
98/96
Sus cebifrons
Sus barbatus
69/39
Sus verrucosus
0.0050
