# Supplementary material for: Bronze Age meat industry: ancient mitochondrial DNA analyses of pig bones from the prehistoric salt mines of Hallstatt (Austria)
Source: BMC Res Notes. 2018 Apr 13;11:243. doi: 10.1186/s13104-018-3340-7 (PMC5899323; doi:10.1186/s13104-018-3340-7)
Supplement: Supplementary file 9 — Additional file 9. Supplementary references. Publications being cited in Additional files 1–9. [file 13104_2018_3340_MOESM9_ESM.pdf]

ADDITIONAL FILE 9: References

Cooper A, Poinar HN (2000) Ancient DNA: do it right or not at all. Science 289:1139

Haring E, Voyta LL, Däubl B, Tiunov MP (2015) Comparison of genetic and morphological characters in fossil teeth of grey voles from the Russian Far East (Rodentia: Cricetidae: *Alexandromys*) Mammal Biol 80:496-504

Kumar S, Stecher G, Tamura K (2016) MEGA7: Molecular Evolutionary Genetics Analysis version 7.0 for bigger datasets. Mol Biol Evol 33:1870-1874

Larson G, Dobney K, Albarella U, Fang M, Matisoo-Smith E, Robins J, Lowden S, Finlayson H, Brand T, Willerslev E, Rowley-Conwy P, Andersson L, Cooper A (2005) Worldwide phylogeography of wild boar reveals multiple centers of pig domestication. Science 307:1618-1621

Lin CS, Sun YL, Liu CY, Yang PC, Chang LC, Cheng IC, Mao SJ. Huang MC (1999) Complete nucleotide sequence of pig (*Sus scrofa*) mitochondrial genome and dating evolutionary divergence within Artiodactyla. Gene 236:107-114

Nei M, Kumar S (2000) Molecular Evolution and Phylogenetics. Oxford University Press, New York.

Watanobe T, Ishiguro N, Okumura N, Nakano M, Matsui A, Hongo H, Ushiro H (2001) Ancient Mitochondrial DNA Reveals the Origin of *Sus scrofa* from Rebun Island, Japan. J Mol Evol 52:281-289

Watt KA (2005) Decontamination techniques in ancient DNA analysis. Master thesis, Simon Fraser University, Burnaby, BC, Canada
